# Sequence of flexion contracture development in the lower limb: a longitudinal analysis of 1,071 children with cerebral palsy

**DOI:** 10.1186/s12891-022-05548-7

**Published:** 2022-07-02

**Authors:** Erika Cloodt, Anna Lindgren, Henrik Lauge-Pedersen, Elisabet Rodby-Bousquet

**Affiliations:** 1 Department of Clinical Sciences Lund, Orthopaedics, Lund University, Lund, Sweden; 2 Department of Research and Development, Region Kronoberg, Växjö, Sweden; 3 Centre for Mathematical Sciences, Lund University, Lund, Sweden; 4 Centre for Clinical Research Västerås, Uppsala University-Region Västmanland, Västerås, Sweden

**Keywords:** Cerebral palsy, Range of motion, Contracture, Joint, Hip, Knee, Foot

## Abstract

**Background:**

To prevent severe contractures and their impact on adjacent joints in children with cerebral palsy (CP), it is crucial to treat the reduced range of motion early and to understand the order by which contractures appear. The aim of this study was to determine how a hip–knee or ankle contracture are associated with the time to and sequence of contracture development in adjacent joints.

**Methods:**

This was a longitudinal cohort study of 1,071 children (636 boys, 435 girls) with CP born 1990 to 2018 who were registered before 5 years of age in the Swedish surveillance program for CP and had a hip, knee or ankle flexion contracture of ≥ 10°. The results were based on 1,636 legs followed for an average of 4.6 years (range 0–17 years). The Cox proportional-hazards model adjusted for Gross Motor Function Classification System (GMFCS) levels I–V was used to compare the percentage of legs with and without more than one contracture.

**Results:**

A second contracture developed in 44% of the legs. The frequency of multiple contractures increased with higher GMFCS level. Children with a primary hip or foot contracture were more likely to develop a second knee contracture. Children with a primary knee contracture developed either a hip or ankle contracture as a second contracture.

**Conclusions:**

Multiple contractures were associated with higher GMFCS level. Lower limb contractures appeared in specific patterns where the location of the primary contracture and GMFCS level were associated with contracture development in adjacent joints.

## Background

Lower limb contractures are often present in children with cerebral palsy (CP). A contracture is defined as reduced joint range of motion (static contracture). The risk of contractures increases with age and severity of the disease [1, 2]. The causes responsible for the development of contractures are not fully understood but are usually referred to spasticity, muscle pathology, muscle weakness, biomechanical alignment, and positioning [3–5]. Contractures are associated with pain and affect the child’s ability to walk, stand, or transfer [6]. Treatment is usually conservative when the contracture first appears and involves physical therapy, orthoses, casting, and treatment for spasticity. Preventive treatment might reduce the need for surgery and should be initiated as early as possible [7].

Children with CP are at risk of developing contractures in multiple joints [8]. It is likely that a single contracture increases the risk of further contractures because of the changes in biomechanical alignment and positioning. Previous studies have shown that hip and knee contractures increase the risk of scoliosis [9, 10]. Therefore, it is important to understand the pattern in which contractures occur to be able to customize effective treatment strategies and prevent severe contractures.

The first contracture to occur in the lower limb varies according to the Gross Motor Function Classification System (GMFCS) level [11]. Children at GMFCS level I or II are most likely to develop an ankle contracture first, whereas children at GMFCS levels III–V are most likely to develop a knee contracture first [12]. To our knowledge, no studies have investigated the association between the location of the first contracture with further contracture development in the lower limb in children with CP.

The aim of this study was to analyze how a hip, knee or ankle contracture affects the time to and sequence of contracture development in adjacent joints in children with CP at GMFCS levels I–V.

## Methods

This was a longitudinal cohort study based on register data from the Swedish Cerebral Palsy Follow-Up Program (CPUP), which includes > 95% of all children with CP in Sweden. Children with at least one flexion contracture in the hip, knee, or ankle joint were included in this study. The study included all measurements reported in the registry since the start of the program in October 1994 until the end of June 2018. All children born in 1990–2018 who were registered in the CPUP before 5 years of age were included. Children registered at 5 years of age or later were excluded. According to CPUP, children are examined every 6 months, once a year, or every other year, depending on their GMFCS level and age [12].

The follow-up includes several variables, such as examination of gross motor function, passive range of motion (ROM), and surgeries reported by the child’s multiprofessional habilitation team and orthopedic department. The full protocol is available at https://cpup.se/in-english/manuals-and-evaluation-forms/. The examiners have access to the child’s previous measurements. The CPUP registry has a yearly reporting rate of 90–95%.

Gross motor function was classified as levels I–V according to the expanded and revised version of the GMFCS [13]. The GMFCS level from the child’s most recent visit were used. Passive ROM was measured using a universal goniometer and standardized positions, and included hip extension (Thomas test), knee extension, and ankle dorsiflexion (knee extended). The range of motion was rounded to the nearest 0 or 5 degrees according to the manual. A contracture was defined as a flexion contracture of at least 10° of the hip and knee or at least 10° plantar flexion of the ankle. The primary outcome was the onset and location of a second contracture.

For children with unilateral CP, only the affected leg was included in the analyses. Children included at baseline had not received lower limb surgeries, selective dorsal rhizotomy operation or an intrathecal baclofen pump before their first hip, knee or ankle contracture occurred. Each leg that underwent soft tissue or bone surgery any time after enrollment, that potentially could influence the risk of further contracture development, was censored at the date of surgery. Examples of reported soft tissue surgeries were adductor tenotomy, hamstring or Achilles tendon lengthening, and tendon or muscle transfer. Examples of reported bone surgeries were osteotomy, physiodesis, or arthrodesis [12]. Both legs were censored from the analysis at the date of a selective dorsal rhizotomy or insert of an intrathecal baclofen pump, or if information about which leg was operated on was missing. Surgery to the upper extremity or the spine, treatment with botulinum toxin injection, oral baclofen medication, orthotics, or serial casting were not censored, and the data for children who received these treatments were retained in the analyses.

### Statistical analysis

All legs with a contracture were followed up individually from the date of the first contracture until the development of the second contracture, the last examination, or date of surgery. The time from first to second contracture was analyzed, for each leg for each child, using the Cox proportional-hazards model, adjusting for GMFCS level. The model assumptions for proportional hazards was tested and fulfilled. This was done first for all legs and then separately according to the joint where the first contracture occurred. Legs with more than one contracture measured at baseline were included in the descriptive analyses (Tables 1 and 2) and when analyzing the time between contractures. They were however excluded when analyzing the sequences of contracture development (Table 3). The chi-squared test was used to analyze in which joint the second contracture occurred according to the GMFCS level; this analysis was performed separately for joints affected by a baseline contracture of the hip, knee, or ankle [14]. The p-for-trend was calculated using logistic regression to analyze systematic, linear, associations of contracture development in these joints relative to the GMFCS level. IBM SPSS Statistics (version 26.0; IBM Corp, Armonk, NY, USA) and R 3.6.1 (R Foundation for Statistical Computing, Vienna, Austria) were used in the statistical analyses. Categorical variables were described in frequency (n) and percentage (%).

**Table 1:** Number of legs and involvement for children at GMFCS level I-V at baseline

GMFCS level	Children N (%)	Legs N (%)	Involvement	
			**Unilateral CP Legs N (%)**	**All other subtypes Legs N (%)**
I	185 (17)	212 (13)	115 (54)	97 (46)
II	206 (19)	269 (16)	68 (25)	201 (75)
III	156 (15)	249 (15)	14 (6)	235 (94)
IV	234 (22)	405 (25)	8 (2)	397 (98)
V	290 (27)	501 (31)	0 (0)	501 (100)
Total	1,071 (100)	1,636 (100)	205 (13)	1431 (87)

*GMFCS* Gross Motor Function Classification System

**Table 2:** Location of the baseline contracture, prevalence of a second contracture during follow-up and number of limb- years for children at GMFCS I-V

**GMFCS level**	**Location of the first contracture**				**Second contracture**		**Limb-years (mean)**
	**Legs N (%)**				**Legs N (%)**		
	**Hip**	**Knee**	**Ankle**	** > 1 joint**		> 1 joint	
I	41 (19)	83 (39)	78 (37)	10 (5)	53 (25)	2 (4)	899 (4.2)
II	43 (16)	98 (36)	112 (42)	16 (6)	101 (38)	2 (2)	989 (3.7)
III	40 (16)	124 (50)	66 (27)	19 (8)	115 (46)	3 (3)	747 (3.0)
IV	58 (14)	210 (52)	90 (22)	47 (11)	203 (50)	8 (4)	1,212 (3.0)
V	77 (15)	294 (59)	90 (18)	40 (8)	248 (50)	9 (4)	1,256 (2.5)
Total	259 (16)	809 (49)	436 (27)	132 (8)	720 (44)	24 (3)	5,103 (3.1)

*GMFCS* Gross Motor Function Classification System

**Table 3:** Results of the chi-squared and p-for-trend analyses of the associations between each baseline contracture and the location of a second contracture during follow-up at each level I to V of the Gross Motor Function Classification System (GMFCS) for legs that developed > 1 lower limb contracture. Only legs with one single baseline and one single follow up contracture were included

First contracture	GMFCS level	Legs N	Second contracture			*p* value
			**Hip N (%)**	**Knee N (%)**	**Ankle N(%)**	
**Hip joint**						0.002^a^
	**I**	8	**–**	5 (63)	3 (37)	< 0.001^b^
	**II**	21	**–**	10 (48)	11 (52)	
	**III**	19	**–**	12 (63)	7 (37)	
	**IV**	28	**–**	22 (79)	6 (21)	
	**V**	45	**–**	40 (89)	5 (11)	
	**Total**	121	**–**	89 (74)	32 (26)	
**Knee joint**						0.003^a^
	**I**	15	4 (27)	**–**	11 (73)	< 0.001^b^
	**II**	32	7 (22)	**–**	25 (78)	
	**III**	54	25 (46)	**–**	29 (54)	
	**IV**	80	36 (45)	**–**	44 (55)	
	**V**	111	65 (59)	**–**	46 (41)	
	**Total**	292	137 (47)	**–**	155 (53)	
**Ankle joint**						< 0.001^a^
	**I**	18	3 (17)	15 (83)	–	0.59^b^
	**II**	30	10 (33)	20 (67)	–	
	**III**	20	3 (15)	17 (85)	–	
	**IV**	40	7 (17)	33 (83)	–	
	**V**	43	9 (21)	34 (79)	–	
	**Total**	151	32 (21)	119 (79)	–	

^a^chi-squared test

^b^ *p*-for-trend analysis

## Results

In total 1,071 children with a lower limb contracture and at least one examination after their initial contracture were included at baseline. The analysis was based on 1,636 legs followed up for an average of 4.6 years (range 0–17 years) after the onset of the first contracture (Table 1). The distribution of the primary contracture varied between GMFCS levels, and multiple contractures were most common at higher GMFCS levels. Within the follow-up period, 916 of the legs (56%) did not develop further contractures, while 720 legs (44%) developed additional contractures (Table 2).

**Fig. 1:**
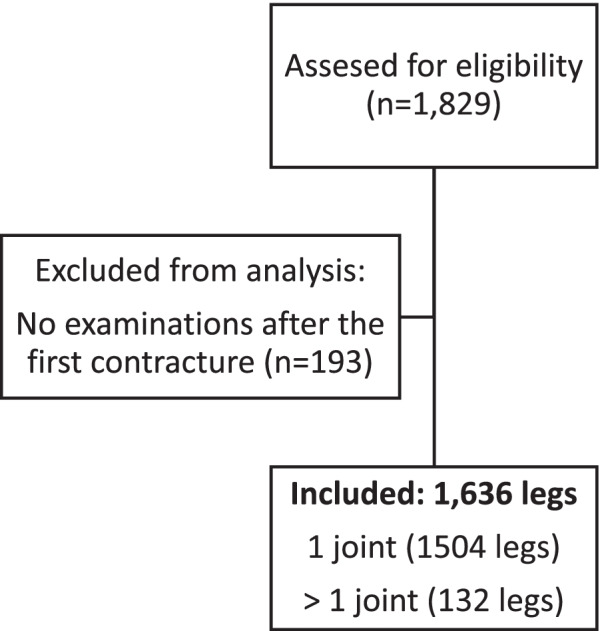
Flowchart of the number of legs included in and excluded from the analyses

In total 132 legs had more than one contracture occurring at baseline. These were counted multiple times; both contractures occurring simultaneously were treated as the first contracture and the potentially following contracture(s) were treated as the second (or third) contracture (Fig. 1). Lower limb surgery was performed on 408 legs (25%) during the follow-up period and were censored from the analysis at the date of surgery. The number lost to follow-up was negligible.

**Fig. 2:**
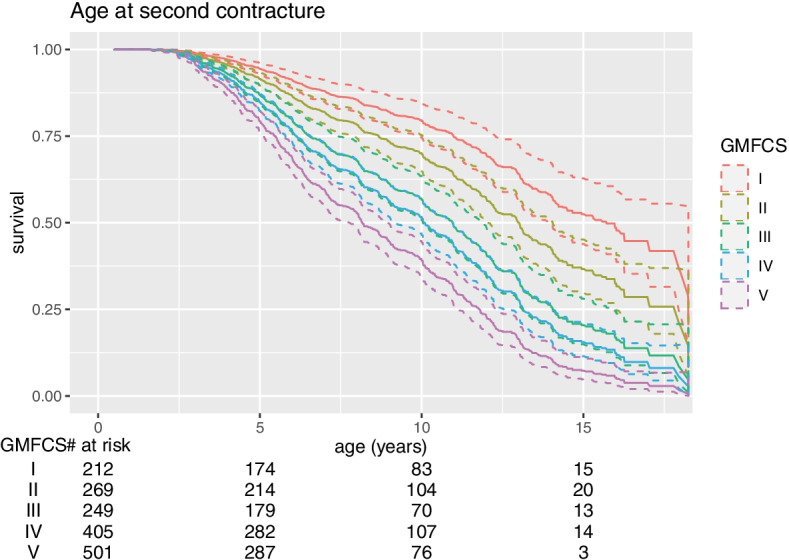
Age at the development of a second contracture for children at GMFCS level I to V

**Fig. 3:**
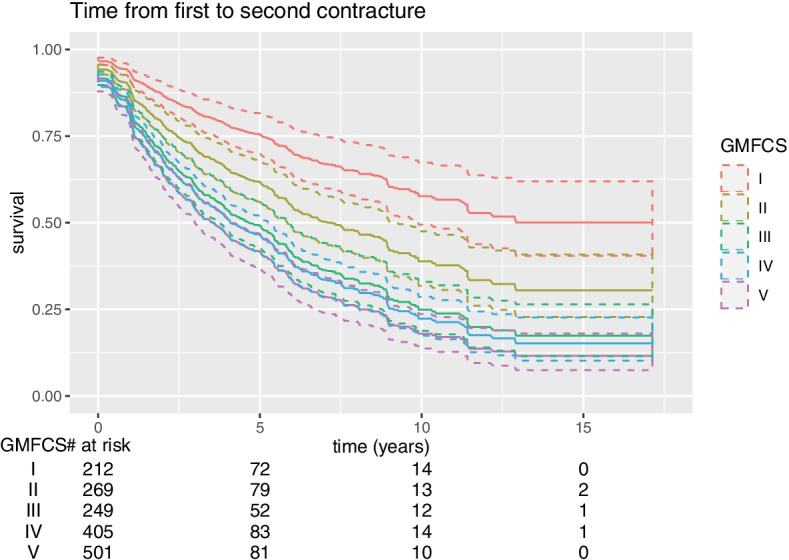
Time in years from the first contracture to the development of a second contracture for children at GMFCS level I to V

The children’s median age for a reported second contracture was 10.8 years. The risk of a second contracture increased with older age and higher GMFCS level (Fig. 2). The second contracture occurred after an average of 5 years from the first contracture (Fig. 3).

**Fig. 4:**
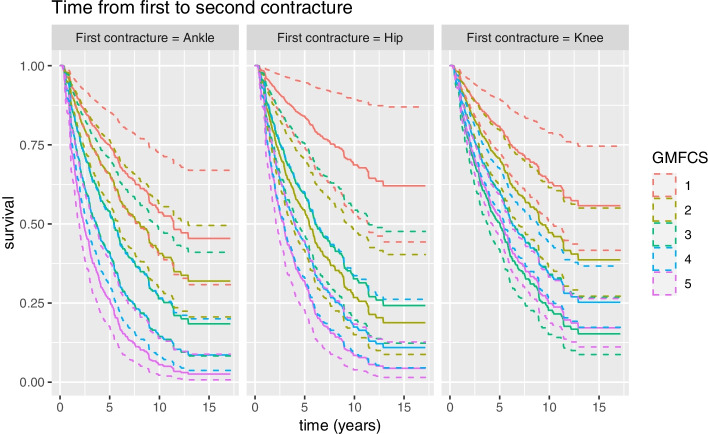
Time to the development of a second contracture based on the location of the first contracture for children at GMFCS I to V

The time from the first to second contracture varied depending on the location of the first contracture. Legs with a primary hip or ankle contracture developed a second contracture earlier than those with a primary knee contracture (Fig. 4).

Legs with more than one baseline contracture were not included in the following analyses.

When analyzing legs with one single baseline contracture and one single follow-up contracture, hip contracture was the primary contracture in 259 legs (Table 3). A second contracture developed during follow-up in 121 (47%) of these legs, most commonly in the knee (74%) and less frequently in the ankle (26%). These results were dependent on the GMFCS level; children at GMFCS level II were more likely to develop an ankle contracture after the hip contracture, whereas those at GMFCS levels I or III–V were more likely to develop a knee contracture after the hip contracture.

A knee contracture was the primary contracture in 809 legs. A second contracture developed in 297 (37%) of these legs with an even distribution between hip (47%) and ankle (53%). A primary knee contracture was more likely to be followed by an ankle contracture for children at GMFCS level I-IV whereas a hip contracture was more likely to be the second contracture in children at GMFCS level V.

An ankle contracture was the first contracture to occur in 436 legs, and a second contracture developed in 151 (35%) of these legs, with more contractures affecting the knee joint (79%) and fewer affecting the hip joint (21%). Having an ankle contracture at baseline increased the risk of developing a knee contracture regardless of GMFCS level (Table 3).

## Discussion

We found that lower limb contractures appeared in specific patterns. A hip or ankle contracture primarily affected the knee joint, whereas a knee contracture affected both the hip and the ankle. Multiple contractures were most common at higher GMFCS levels. Of the children, 25% at GMFCS level I and 38% at level II developed more than one contracture during the follow-up compared with 50% of children at GMFCS IV and V. This corresponds with previous findings [2] that children at GMFCS IV and V are most likely to develop lower limb contractures.

The median age for developing a second contracture was 10 years. The second contracture was reported after a median of 5 years from the first contracture. The key problems in children with CP are muscle pathology, loss of muscle control, abnormal muscle tone and muscle imbalance and these are associated with secondary problems, such as short muscles and contractures during childhood and growth. The secondary problems lead to tertiary abnormalities because the child must use compensatory strategies. It is important to understand the natural history and multifactorial pathology of contracture development [15, 16].

This study showed that the most common pattern in which the contractures appeared was a knee contracture at baseline followed by a hip or ankle contracture. The location of the second contracture varied with GMFCS level, and children at GMFCS level V were more likely to develop a hip contracture secondary to the knee contracture. Children with bilateral CP are, in general, more affected by deformities of the hip, whereas those with unilateral CP have more involvement of the ankle [17]. When Pettersson et al. [10] created a risk score for the development of scoliosis before 16 years of age in children with CP, limited knee extension was identified as an independent predictor. In supine lying, limited knee extension may force the hips into flexion and gravity is likely to tilt the legs to one side, causing the windswept position [18]. That may be one reason why a knee contracture increases the risk of a hip contracture. Children at GMFCS level IV or V are mostly nonambulatory and spend more time sitting with flexed knees and hips. This could also contribute to the sequence in which the contracture presents. Children at higher GMFCS levels have a high prevalence of postural asymmetries in sitting and lying, and they have difficulties to change position [19]. A knee contracture also increases the risk of an ankle contracture, possibly because of the gastrocnemius muscle, which is involved in both knee flexion and plantar flexion.

For ambulatory children with bilateral CP, a knee contracture may force them to walk in apparent equinus because of the lever arm dysfunction, which could lead to an ankle contracture. It is important to recognize apparent equinus when children walk on their toes because of the flexion contracture of the knee and not because of an ankle contracture [20]. For children with unilateral CP, a knee contracture can cause a limb-length discrepancy that makes them use toe walking as a compensatory strategy [21].

We found that children who developed multiple contractures and had an ankle contracture at baseline, were most likely to develop a knee contracture during the follow-up independent of their GMFCS level. In Sweden, children with limited dorsiflexion are usually treated with ankle–foot orthoses [22]. Most of these children use their orthoses during sleep. By preventing plantar flexion of the foot, a short gastrocnemius may force the knee joint into flexion and the child to sleep with flexed knees, which could cause a knee contracture over time [16]. This may lead to a vicious cycle with more time in sitting and less time in standing.

Limited dorsiflexion and equinus in children with unilateral CP may also compensate for the leg-length discrepancy by causing them to walk with a flexed knee on the longer leg [21]. In Sweden, botulinum toxin A injections are a common treatment for reduced ROM caused by increased muscle tone in the plantar flexors [23]. Together with isolated lengthening of the Achilles tendon, excessive injection of botulinum toxin A into the calf muscles can cause crouch gait, which is likely to persist and deteriorate over time with increased knee flexion [20].

There are several limitations to our study. Even though all subtypes were included, only spastic unilateral CP was identified to select the affected leg. The examinations started in 1994, prior to the introduction of the GMFCS. Therefore, we used the GMFCS level from the most recent report. However, the GMFCS levels show stability over time [24]. The contractures were recorded by goniometric measurement, which is a standard measurement in clinical settings but whose reliability varies according to the joint and position [25–27]. The results of our study were based on repeated measurements by many different examiners, and this may have introduced information bias or measurement errors that could influence the survival analysis. However, this bias has been shown to be small when differences between groups are small in terms of hazard ratios [14]. We validated the data for incongruent measurements to reduce the risk of extreme outliers caused by errors, e.g. clear reporting errors such as ROM of 360 degrees. The examiners involved in the CPUP are encouraged to practice the standardized measurements in the program, which has been shown to be important for reliability [28].

Another limitation was the cutoff value of a contracture because –10° in the hip, knee, and ankle joint can also affect the order in which a contracture appears. Full passive range of motion allows more extension of the hip and ankle joints compared with the knee joint. However, we could not find any literature supporting a cutoff exceeding 0° in any of the joints being considered a contracture. The statistical analyses were also run with a cutoff value of 0° and produced similar outcomes. There are few reference values for passive ROM in children with CP and no standard definition of what ROM is considered a contracture [29, 30]. Reference values for typically developing children are not representative when describing ROM in children with CP, who have reduced knee and ankle extension compared with hip extension [29]. We chose –10° as the cutoff for all joints since these values are often applied in clinical practice as severely affecting biomechanical alignment, gait, function and positioning. Hip extension less than 0° affects mobility and function in ambulatory children with CP, and a hip flexion contracture of -15° significantly decreases the physical functioning [31]. A knee flexion contracture of -10° increase the risk for crouch gait, and -10° of plantarflexion is the minimum required to significantly change kinematics and kinetics[30, 32, 33].

A further limitation of this study was the age of the children censored because of surgery. In the CPUP follow-up program, children at risk for hip displacement (migration percentage > 40) have their first soft tissue surgery of the hip early at a median age of 4 years. We wanted to follow the natural history of contracture development as much as possible, and it is likely that surgery of a joint affects the ROM both in the specific joint and the adjacent joints. Therefore, we censored legs at the date of surgery. This may have affected the results, especially for children at GMFCS level IV or V, who are more likely to have a surgery at a younger age. It is also possible that this decision led to underestimation of the influence of hip contracture on other joints. This may also partially explain why a hip flexion contracture was the least common contracture in our study. However, loss of hip extension seems less common than loss of knee extension and ankle dorsiflexion in children with CP at GMFCS I-II [29].

Children who received botulinum toxin treatment were not excluded from analyses and it could be argued that this interfered with our results since this treatment is more common in muscles affecting the knee and ankle than muscles affecting the hip. However, given the large number of children having this treatment[23] and the fact that the effect is not permanent, these children were included. Most children followed in CPUP receives ongoing interventions such as physiotherapy and treatment with orthoses. This should be taken under consideration when translating the results to other groups. This study covers a period of 24 years and interventions have changed during this time from more hands-on therapy to focus on participation and activities in daily life.

Contracture development starts early during the rapid growth in childhood and the risk of fixed contractures increases with age [2]. To prevent decompensation due to fixed deformities, clinicians should treat contractures as early as possible [34]. By monitoring children with CP, and treating contractures when they appear, with e.g. physiotherapy and orthoses, it may be possible to avoid the need for major surgery [7]. In the clinical setting, it is important to recognize how the sequence of contractures is likely to occur and to initiate prophylactic treatment of adjacent joints e.g. consider the knee joint when introducing Ankle–Foot Orthoses. For the orthopedic surgeon, knee contracture might indicate the need for closer monitoring of the hip to avoid dislocation [35].

## Conclusions

Development of a second contracture is a common problem in children with CP and associated with higher GMFCS level. The time and sequence of secondary contracture development in the lower limbs are influenced by the location of the first contracture and GMFCS level. The second contracture primarily develops in the adjacent joint.

Lower limb contracture tends to appear in specific patterns, in which the location of the primary contracture affects the later development of secondary contracture in the adjacent joints. This should be considered when treatment strategies to prevent contractures are being formed.

## Data Availability

The dataset analyzed for this study are available in the CPUP registry. Permission to access data after ethical approval is granted by KVB Region Skåne. Requests to access the dataset should be directed to https://vardgivare.skane.se/kompetens-utveckling/forskning-inom-region-skane/utlamnande-av-patientdata-samradkvb/.

## Abbreviations

CP: Cerebral Palsy
CPUP: Cerebral Palsy Follow-up Program (Sweden)
GMFCS: Gross Motor Function Classification System
ROM: Range of Motion
